# Loss of miR-132/212 Has No Long-Term Beneficial Effect on Cardiac Function After Permanent Coronary Occlusion in Mice

**DOI:** 10.3389/fphys.2020.00590

**Published:** 2020-06-16

**Authors:** Zhiyong Lei, Juntao Fang, Janine C. Deddens, Corina H. G. Metz, Esther C. M. van Eeuwijk, Hamid el Azzouzi, Pieter A. Doevendans, Joost P. G. Sluijter

**Affiliations:** ^1^Department of Cardiology, Experimental Cardiology Laboratory, Division Heart and Lungs, University Medical Center Utrecht, Utrecht, Netherlands; ^2^National Heart Institute, Utrecht, Netherlands; ^3^Central Military Hospital Utrecht, Utrecht, Netherlands; ^4^UMC Utrecht Regenerative Medicine Center, Circulatory Health Laboratory, University Utrecht, University Medical Center, Utrecht, Netherlands

**Keywords:** miR-132/212, myocardial infarction, cardiac function, adverse cardiac remodeling, permanent coronary occlusion

## Abstract

**Background:** Myocardial infarction (MI) is caused by occlusion of the coronary artery and induces ischemia in the myocardium and eventually a massive loss in cardiomyocytes. Studies have shown many factors or treatments that can affect the healing and remodeling of the heart upon infarction, leading to better cardiac performance and clinical outcome. Previously, miR-132/212 has been shown to play an important role in arteriogenesis in a mouse model of hindlimb ischemia and in the regulation of cardiac contractility in hypertrophic cardiomyopathy in mice. In this study, we explored the role of miR-132/212 during ischemia in a murine MI model.

**Methods and Results:** miR-132/212 knockout mice show enhanced cardiac contractile function at baseline compared to wild-type controls, as assessed by echocardiography. One day after induction of MI by permanent occlusion, miR-132/212 knockout mice display similar levels of cardiac damage as wild-type controls, as demonstrated by infarction size quantification and LDH release, although a trend toward more cardiomyocyte cell death was observed in the knockout mice as shown by TUNEL staining. Four weeks after MI, miR-132/212 knockout mice show no differences in terms of cardiac function, expression of cardiac stress markers, and fibrotic remodeling, although vascularization was reduced. In line with these *in vivo* observation, overexpression of miR-132 or miR-212 in neonatal rat cardiomyocyte suppress hypoxia induced cardiomyocyte cell death.

**Conclusion:** Although we previously observed a role in collateral formation and myocardial contractility, the absence of miR-132/212 did not affect the overall myocardial performance upon a permanent occlusion of the coronary artery. This suggests an interplay of different roles of this miR-132/212 before and during MI, including an inhibitory effect on cell death and angiogenesis, and a positive effect on cardiac contractility and autophagic response. Thus, spatial or tissue specific manipulation of this microRNA family may be essential to fully understand the roles and to develop interventions to reduce infarct size.

## Introduction

Although the mortality rate of myocardial infarction (MI) in patients is going down due to recently developed post-infarction treatments and secondary prevention, MI is still one of the leading causes of mortality (Mozaffarian et al., 2015). 12.5% of patients that suffered a MI with ST-segment elevation may die within 6 months (Steg et al., 2012), suggesting that novel effective treatments are still required.

microRNAs are small non-coding RNAs that play essential roles in cardiac development, and dysregulation of microRNAs promote the pathological progression of many cardiac diseases. Therapeutic interventions are beneficial to slow down the myocardial pathological progression (Bonauer et al., 2009; Montgomery et al., 2011; Grueter et al., 2012; Hullinger et al., 2012; Wahlquist et al., 2014), and therefore microRNAs have been considered as promising therapeutic targets for cardiovascular diseases (van Rooij, 2014; van Rooij and Kauppinen, 2014).

The miR-132/212 family plays essential roles in maintaining physiological function and pathological disease progression of the cardiovascular system. The expression level of this family goes upon angiogenic stimulation, including hypoxia (Burek et al., 2019) or loss-of-VHL (Lei et al., 2020) and during hypertrophic growth upon Angiotensin II treatment (Eskildsen et al., 2013). Loss of miR132/212 shows impaired angiogenesis response in hindlimb ischemia model (Lei et al., 2015) and overexpression of miR132/212 enhance neovascularization. They are also reported to be upregulated in the failing human heart, where they play a detrimental role in the regulation of cardiomyocyte contractility and the cardiac hypertrophy in hypertension-induced heart failure models (Ucar et al., 2012). However, the regulation and biological function of this family in the response to a MI has never been investigated. We, therefore, used the miR-132/212 genetic knockout (KO) mice and induced MI by permanent occlusion of the coronary artery to explore the functional effects on cardiac function compared to wild-type controls.

## Materials and Methods

### Generation and Genotyping of miR-132/212 KO Mice

**FIGURE 1:**
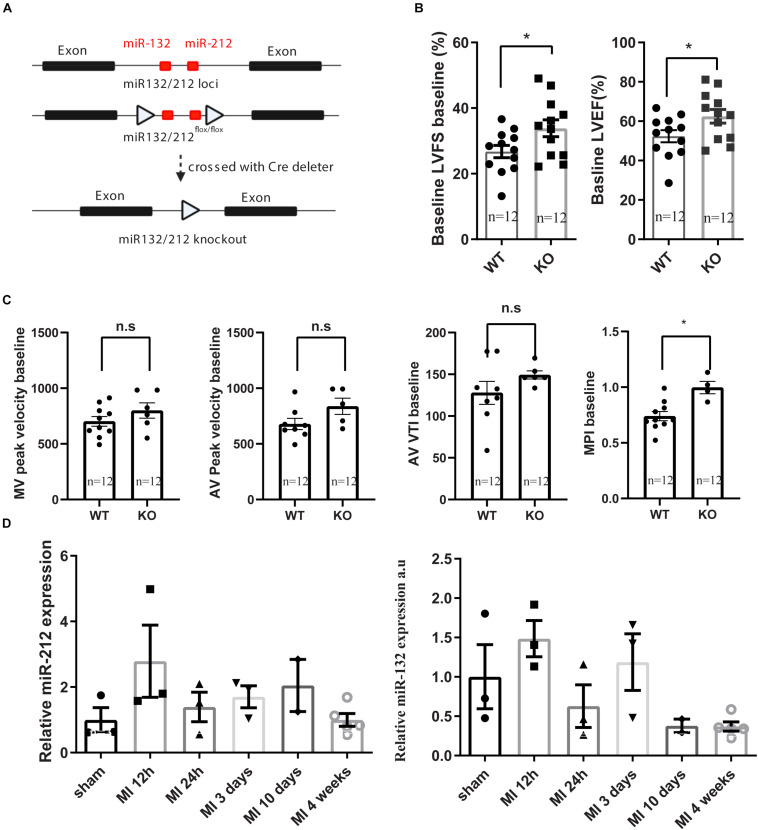
Generation of miR132/212 KO mice and base line characterization the cardiac function of the knockout mice. **(A)** Gene targeting strategy of miR132/212. **(B,C)**. base line characterization the cardiac function of the knockout mice. **(D)** expression of miR132 and mir212 after myocardial infarction. LVFS: left ventricular fraction shorting; LVEF: left ventricular enjection fraction; MPI: myocardial performance index; MV: Mitral valve; AV: Aortic valve; AV VTI: Aorta velocity time integral.

The miR-132/212 KO mice have been generated as described previously (Kayo et al., 2014). In brief, the miR-132/212^flox/flox^ mice were generated by gene targeting in mouse ES cell from the C57BL/6N background and then crossed with a Cre deleter line to remove the miR-132/212 genome region (see Figure 1A). The resulted miR-212-132^–/–^ line is subsequently maintained in C57BL/6J mice background. To reduce the ES C57BL/6N background, it was back crossed with C57BL/6J for 6 times. All the animal experiment in this study was carried out using age and sex-matched C57BL/6J mice as wildtype (WT) control from the same bread. miR132/212 localizes between the exon1 and exon2 od HIC1 gene. After the removal of the miR-132/212 region, HIC1 and neighboring gene expression is not altered. For genotyping, DNA samples were obtained by ear clipping and used in a GC-Rich PCR kit (Roche, cat. 12140306001) with miR-132/212 primers as shown in Table 1. PCR products were revealed on a 1% agarose gel: WT genotype display a predicted band at 1076 bp and the KO genotype at 392 bp.

**TABLE 1:** Cardiac function analysis of miR-132/212 knockout and WT mice by echocardiography at baseline: LVFS; LVEF; myocardial performance index (MPI), peak velocity of MV and AV.

	Base line		1 week after MI		2 weeks after MI		4 weeks after MI	
	WT (*n* = 12)	KO (*n* = 12)	WT (*n* = 6)	KO (*n* = 5)	WT (*n* = 6)	KO (*n* = 5)	WT (*n* = 5)	KO (*n* = 5)
Heart_Rate	460.9 ± 10.4	485.4 ± 8.0	643.49 ± 69.1	501.92 ± 11.23	456.2 ± 21.1	398.7 ± 12.9	614.8 ± 96.3	506.6 ± 16.2
Diameter_systolic	2.8 ± 0.1	2.4 ± 0.1	4.18 ± 0.28	3.86 ± 0.25	4.4 ± 0.4	5 ± 0.4	5.1 ± 0.2	5.1 ± 0.5
Diameter_diastolic	3.8 ± 0.1	3.7 ± 0.1	4.83 ± 0.24	4.51 ± 0.14	5.1 ± 0.3	5.5 ± 0.4	5.7 ± 0.2	5.6 ± 0.5
Volume_systolic	30.2 ± 2.6	22.6 ± 3.1	80.59 ± 12.49	66.18 ± 9.48	94.5 ± 15.5	123.9 ± 21.9	126.9 ± 8.7	131.2 ± 24.3
Volume_diastolic	63.0 ± 3.1	57.8 ± 4.2	110.96 ± 12.31	93.7 ± 6.65	124.3 ± 15.7	151.6 ± 24.1	159.1 ± 11.4	160.1 ± 29.4
Stroke_volume	32.8 ± 2.6	35.2 ± 2.4	30.38 ± 3.08	27.52 ± 5.31	29.8 ± 2.6	27.6 ± 6.7	32.3 ± 4.8	28.9 ± 9.5
Ejection_fraction	52.4 ± 3.1	62.5 ± 3.5*	29.06 ± 3.67	30.61 ± 6.72	26.8 ± 5.6	19 ± 5.1	20.2 ± 2	18.8 ± 4.6
Fraction_shortening	26.7 ± 1.9	33.8 ± 2.6*	13.69 ± 1.81	14.68 ± 3.54	12.8 ± 3	8.9 ± 2.5	9.3 ± 1	8.7 ± 2.2
Cardiac_output	15.3 ± 1.6	17.1 ± 1.3	20.13 ± 3.73	14.04 ± 3.03	13.5 ± 1.1	10.9 ± 2.5	19.2 ± 2.8	15 ± 5.4

### LAD Ligation and Echocardiography

This study was approved by the Animal Ethical Experimentation Committee (DEC. 2013.II.02.019, Utrecht University) and was carried out in accordance with the Guide for the care and use of Laboratory Animals.

Myocardial infarction (MI) was induced by ligation of the Left anterior descending artery (LAD) and applied on 10–12 weeks old WT (C57B6) and miR-132/212 KO mice, as described previously (Grundmann et al., 2011). In brief, mice were anesthetized with fentanyl (0.05 mg/kg), midazolam (5 mg/kg) and medetomidine (0.5 mg/kg) by intraperitoneal injection and surgical procedures were performed under sterile conditions. LAD was ligated just below the left atrial appendage with an 8–0 Ethilon monifil suture. The chest was then closed and animals received atipamezole (2.5 mg/kg) and flumazenil (0.5 mg/kg) to recover quickly. Temgesic (0.1 mg/kg) was given every 8 h after surgery for 6 times to reduce discomfort. Cardiac function was assessed with echocardiography (Vevo^®^ 2100 System, Visualsonics) and analyzed with Vevo2100-1.6.0 (Visualsonics) before and after the surgical procedure (days 0, 7, 14, and 28). During the procedure, the animals were kept under 2% isoflurane anesthesia and the body temperature was strictly maintained between 36.5–37.5°C. To terminate the mice, mice were anesthetized by an overdose anesthesia with fentanyl (0.1 mg/kg), midazolam (10 mg/kg), and medetomidine (1 mg/kg) by intraperitoneal injection.

### Neonatal Rat Cardiomyocytes Isolation and Hypoxia Treatment

Neonatal rat cardiomyocytes isolation was performed with Pierce Primary Cardiomyocyte Isolation Kit (Life Technologies, Cat. 88281) following manufacture’s instruction. In brief, neonatal rat hearts were collected within 3 days after birth. After washing with ice cold Hank’s Balanced Salt Solution (HBSS) (Gibco), hearts are cut into small pieces before enzymatic digestion for 35 min. After digestion, pieces are washed with cold HBSS once again and disassociated with cardiomyocyte culture medium with 10% FBS and single cells generated by filtrating over a 40 μm filter to remove undigested tissue. After centrifuge, cells were re-suspended in culture medium with 10% FBS and seeded at 2.5 × 10^5^cells/cm^2^. The next day, cells were transfected with microRNA mimics mirVana miRNA mimic scramble control (4464085), hsa-miR-132-3p mimics (MC10166), hsa-miR-212-3p mimics (MC10340) with RNAiMAX (Life Technologies) at 50nM following manufacture’s instruction. Six hours after transfection, medium was replaced with fresh DMEM medium with 10% FBS containing 1× Cardiomyocyte Growth Supplement. Forty-eight hours after transfection, cells were transferred to a hypoxia chamber with 5% CO_2_ and 1% O_2_ for 24 h. Then cells were fixed with 4% PFA for 15 min before TUNEL staining.

### TUNEL Staining

Twenty-four hours after MI surgery (*n* = 4/group), mice were terminated. Hearts were explanted, rinsed and fixed with 0.2 PFA in 15% sucrose at 4°C overnight before cryopreservation with Tissue Tek for sectioning. Ten μm cryosections were prepared. For TUNEL staining, sections were first dried for 10 min at room temperature, then digested with 5 μg/ml proteinase K (cat. Roche) for 20 min at 37°C. Sections were subsequently used for TUNEL staining (*In situ* Death Detection Kit, Cat. 1684795, Roche) following manufacturer’s instructions. After TUNEL, sections were counterstained with Hoechst for nuclei and Troponin for cardiomyocytes. Images were taken and analyzed by a blinded investigator with Cellsens imaging system at 20× magnification. TUNEL staining for hypoxia treated Rat neonatal cardiomyocytes (RNCM) was performed in a similar approach without Proteinase K treatment.

### Infarct Size Quantification

Twenty-four hours after MI surgery (*n* = 6/group), infarct size (IS) was determined as a percentage of the area at risk (AAR). Four% Evans Blue solution was injected via the thoracic aorta and hearts were explanted, rinsed and filled with paper before placement in −20°C freezer for 1 h. Hearts were subsequently sliced into 1mm cross sections and incubated with 1% triphenyltetrazolium chloride (TTC, Sigma) for 1 h at 37°C, then fixed with formaldehyde 4% for 15 min. Images from both sides of the cardiac sections were taken sequentially from apex to atrium. IS, AAR and left the ventricular area were measured with Photoshop and reconstructed as previously described in ImageJ (Koudstaal et al., 2015).

### Lactate Dehydrogenase and Troponin Measurement

After termination at 24 h post-MI, blood samples (*n* = 4/group) were collected by cardiac puncture. Samples were centrifuged at 12,000×*g* for 10 min and cleared plasma then transferred to another tube. For total lactate dehydrogenase assay (LDH), 10 μl of plasma was used to determine total LDH concentration using the Toxicology Assay Kit (Cat. TOX7-1KT, Sigma) according to manufacturer’s instructions with an arrayscan at 492 nM (Thermo Fisher). cTnI levels were measured by ELISA (Synchron Lxi 725 integrated clinical chemistry, Beckman Coulter) in the Laboratorium Klinische Chemie en Hematology (LKCH) of UMC Utrecht as previously described (Oerlemans et al., 2012).

### Histological Analysis and Immunohistochemical Staining

28 days after MI (long-term group, *n* = 6/group), mice were terminated. Hearts were explanted, rinsed and fixed with 0.2 PFA in 15% sucrose at 4°C overnight before cryopreservation with Tissue Tek for sectioning. HE staining and Picrosirius red staining were performed for morphological and fibrotic remodeling assessment, respectively, as described before (Timmers et al., 2008). For evaluation of large vessels, sections were first blocked with 2% BSA for 30 min, FITC-labeled anti-αSMA antibody was applied for 1 h at room temperature. After incubating the slides with 1 mg/ml Hoechst to visualize the nuclei, sections were mounted in fluoromount G (Southern Biotech). The complete sections were then scanned for both αSMA and Hoechst channel. Images were analyzed with ImageJ. All the αSMA positive signal larger than 500 arbitrary unit (a.u.) (proximately two nuclei) were considered as a vessel. The vessel coverage was calculated by total vessel area divided by total number of cells and vessel density was calculated by the total number of vessels divided by the total number of the cells.

### RNA Isolation and RT-PCR Analysis

DNA-free RNA was extracted with Tripure (Roche applied science). To perform qPCR for gene expression, RNA is transcribed to cDNA using the iScript cDNA Synthesis Kit (Bio-Rad) according to manufacturer’s instructions, and quantitative real-time PCR was performed on a MyIQ single-color qRT-PCR system (Bio-Rad), as described previously (van Mil et al., 2012). All primers used for qPCR analysis are listed in the Table 1. Mature miR-132 and miR-212 expression levels were measured by TaqMan^®^ MicroRNA Assay following manufactory’s instruction, using U6 as control.

### Statistical Analysis

Data was analyzed using Graphpad Prism 8 and comparisons were performed with *t*-test between two groups. For multiple groups, and time-course measurement, two-way ANOVA were used with *post hoc* Bonferroni correction. Data are presented as mean ± SEM. *p* < 0.05 is considered as significant, labeled with^∗^.

## Results

### miR-132/212 Knockout Mice Show More Damage in the Heart After MI

Consistent with our previous observations, miR-132/212 knockout mice display enhanced cardiac contractile function as shown by higher left ventricular fraction shortening (LVFS), left ventricular ejection fraction (LVEF) and myocardial performance index (MPI) at baseline, as shown in Figures 1B,C.

**FIGURE 2:**
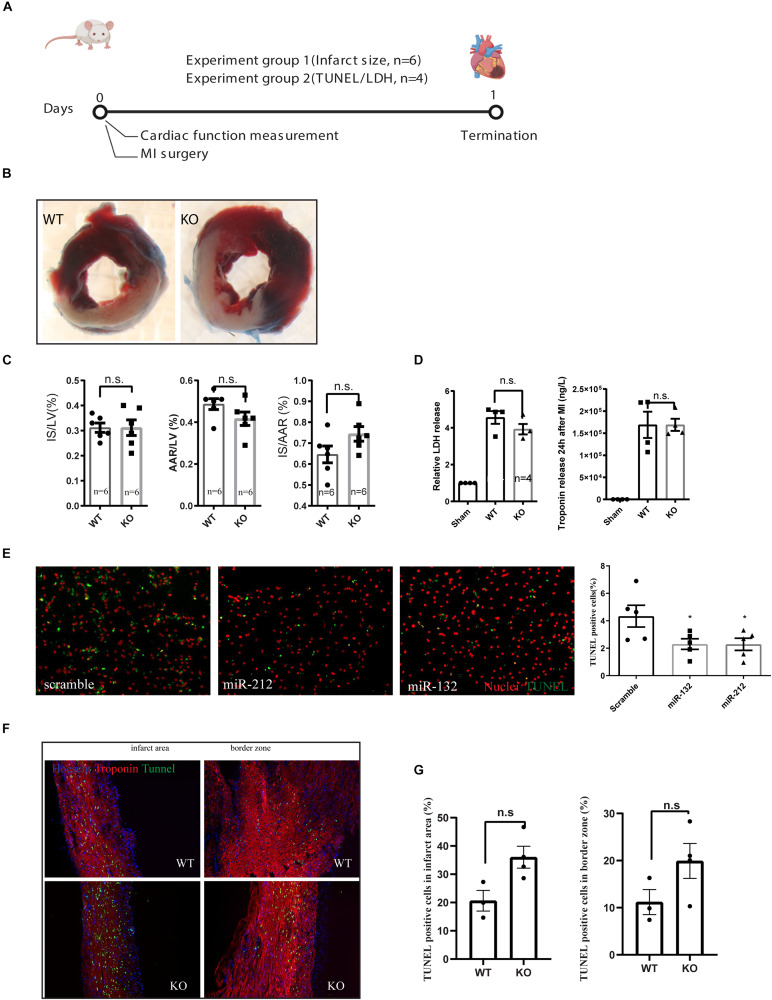
Characterization of cardiac damage after MI by TTC and TUNEL staining. **(A)** Experiment design for short term cardiac damage assessment. **(B)** representative images of WT and KO hearts stained with TTC 24 h after MI. **(C)** Quantification of infarct size (IS), area at risk (AAR) of left ventricle (LV), **(D)** LDH and Troponin release in the serum at 24 h after MI as measured by ELISA. **(E)** representative images of TUNEL staining on rat neonatal cardiomyocytes, transfected with indicated microRNA mimics or scramble controls and treated for 24 h of ischemia and the quantification of TUNEL positive cells. **(F,G)** Representative images of TUNEL staining on the left ventricle 24 h after MI and their quantifications.

For assessing the role of miR-132/212 in the setting of an acute MI, LAD ligations were performed in miR-132/212 knockout and WT control mice Figure 2A. 24 h post-MI, cardiac damage was assessed by TTC staining on cardiac slices from operated WT and KO mice (Figure 2B). No significant differences were observed in infarct size (IS), as measured both in the percentage of LV (IS/LV) and in the percentage of area at risk (IS/AAR). Although KO mice display a trend toward higher IS/AAR (Figure 2C), circulating Lactate Dehydrogenase (LDH) levels and Troponin levels, markers for cardiac damage, did not differ between WT and KO mice (Figure 2D).

To further explore the effect of miR-132/212 in cell death, we overexpressed miR-132 and miR-212 in hypoxic RNCM for 24 h. Cardiomyocytes with overexpression of miR-132 or miR-212 are more resistant to ischemia-induced cell death, as shown by TUNEL staining (Figures 2E,G) indicating that miR-132 and 212 are indeed protective for ischemia in cardiomyocytes.

TUNEL staining was then performed on cross-sections of infarcted hearts 24 h post-MI to determine differences in cell death. A trend to an increased percentage of TUNEL positive cells in the KO mice is observed but did not reach significance in both border zone and infarcted area compared to WT control hearts (Figures 2F,G).

### Loss of miR-132/212 Shows No Benefit in Cardiac Function Preservation or Adverse Cardiac Remodeling

**FIGURE 3:**
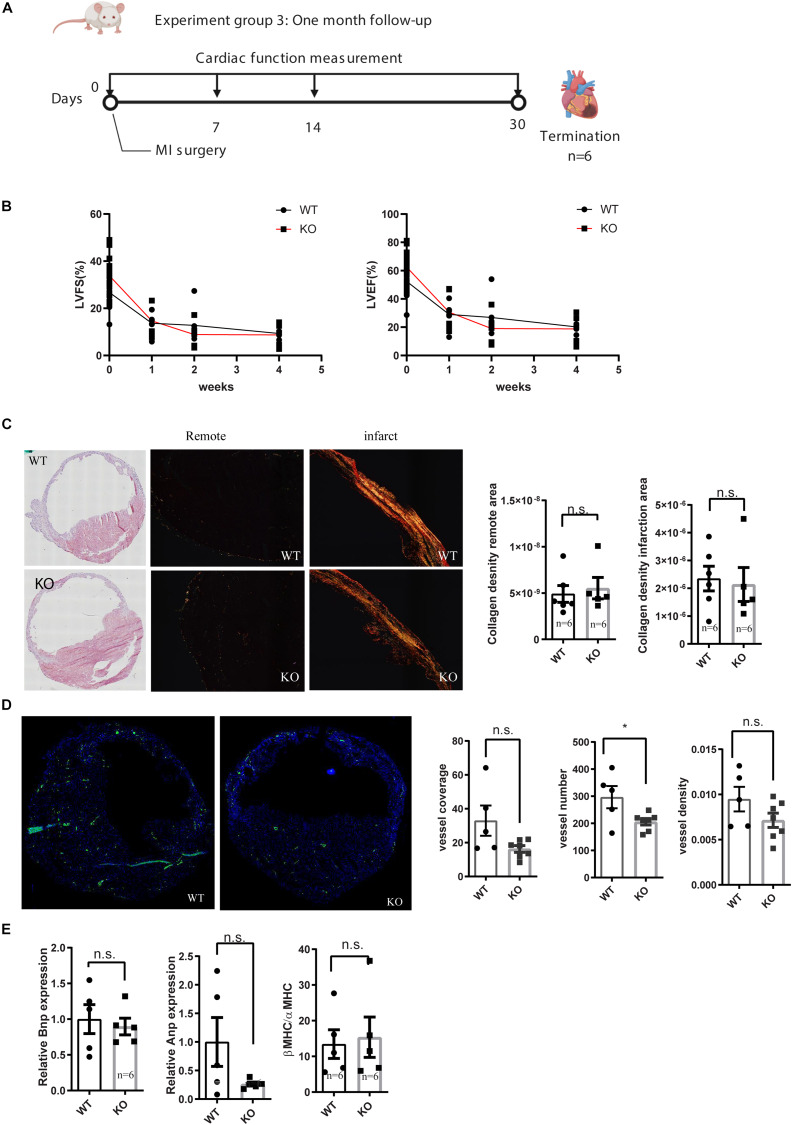
Characterization of cardiac function of miR-132/212 knockout mice by echocardiography 4 weeks after MI. **(A)** experiment design for month cardiac function follow-up after myocardial infarction. **(B)** LVEF as a percentage in WT and KO mice during 4 weeks post- MI. **(B)** LVFS as a percentage in WT and KO mice during 4 weeks post- MI. **(C)** representative images of HE staining of the WT and KO cardiac section 4 weeks post-MI, representative images of Picrosirius red staining for collagen content of the WT and KO cardiac section and quantification of collagen density. **(D)** Representative images of αSMA staining in WT and KO cardiac section and quantification of number of the αSMA positive vessel, normalized vessel coverage and vessel density. **(E)** Molecular characterization of the hearts 4 weeks post-MI by qPCR for cardiac stress markers: Anp, Bnp, and βMHC/αMHC ratio.

To see the long term consequence of miR-132/212 loss post-MI, we exposed another set of mice to MI and followed their cardiac function by echocardiography for 4 weeks Figure 3A. Consistent with the previously observed effect in cell death, we observed that KO mice demonstrated a stronger reduction in cardiac function than WT mice within the first 2 weeks (Figure 3B). However, eventually both WT and KO animals display similar cardiac function at 4 weeks, exemplified by a similar reduction in ejection fraction and fractional shortening (Figure 3B).

After the termination of these mice at 4 weeks post-MI, we further characterized their hearts at the histological and molecular levels. Both WT and KO displayed extensive cardiac remodeling and expansion of the IS (Figure 3C). No differences in fibrotic remodeling, both in the infarct and remote areas could be observed between WT and KO mice (Figure 3C). To assess the stress status of the hearts, we checked the expression of several cardiac stress markers, but no significant difference was detected in Anp, Bnp, nor in the βMHC/αMHC ratios (Figure 3E).

Neovascularization has been shown to play a role in cardiac healing and remodeling after MI (Takeda et al., 2009; Zarrinpashneh et al., 2013) and we have previously observed that miR132/212 did affect the arteriogenic response after hind-limb ischemia. To see if the loss of miR-132/212 could also affect the neovascularization after MI, we stained for αSMA to visualize larger vessels which are mainly responsive for actual blood supply. We observed that KO mice display a lower number of vessels upon MI. There was a trend toward a lower vessel density and vessel coverage rate in the KO mice, but these were not statistically significant (Figure 3D).

## Discussion

In this study, we tested the role of miR-132/212 during myocardial infarction using genetic knockout mouse both on the short and long term post-MI. Four weeks after MI loss of miR-132/212 did not show any differences in the cardiac function or adverse cardiac remodeling. These results indicated that general inhibition of miR-132/212 in the setting of MI has no beneficial effect in the preservation of cardiac function.

Loss of miR-132/212 did not show any beneficial effects on cardiac function at 4 weeks, both on cardiac function as well as histological levels, although ths miRNA family is described to play a role in neovascularization and myocardial contractility regulation. Several distinct mechanisms might explain this observation. First, we and others found that miR-132/212 modulate the Ras-MAPK pathway by synergistically suppressing multiple intrinsic inhibitors of the Ras-MAPK [Rasa1 (Katare et al., 2011), Spred1 and Spry1] and PI3K-AKT pathway (PTEN) in Human Umbilical Vein Endothelial Cells (HUVECs). It also has been shown that miR-132/212 has an anti-apoptotic role by activating the PI3K-AKT pathway in a mouse cardiomyocyte line (Ucar et al., 2012). Thus, miR-132/212 may have a positive effect on cardiomyocyte cell death by directly regulating survival signaling during ischemia. Secondly, miR-132/212 regulates the contractility of the heart. Reducing wall stress after MI, either by mechanically unloading the heart (Kapur et al., 2013) or pharmaceutically by using ACE inhibitors or beta-blockers (Gajarsa and Kloner, 2011), is beneficial to cardiac healing after MI. Therefore we believe that the loss of miR-132/212 enhances both cardiac contractility and increases stress levels post-MI. In this sense, miR-132/212 inhibition may induce more damage following MI, a detrimental effect undesirable in the clinical setting.

Although miR-132/212 plays a protective role in the immediate-early phase post-MI, associated with an early increase in expression 12h post-MI, its expression is decreased at 24 h but increased again in a second wave in later phases but didn’t reach statistical significance Figure 1D. The upregulation of this miR-132/212 in the later phase is potentially impairing cardiac contractility and the associated autophagic response (Ucar et al., 2012). It remains to be tested if inhibition of miR-132/212 at later stages after MI may still help to maintain cardiac contractile function while keeping or even increasing the expression of miR-132/212 in the early phase. For that purpose, conditional knockout mice, antagomiRs or better targeting of therapeutics should be used (Kwekkeboom et al., 2014), and at least a substantial amount of viable myocardium has to be preserved before any effect of miR-132/212 inhibition can be observed. Nevertheless, our results demonstrate yet another example for multifunction properties of a single microRNA, emphasizing that a spatial and/or tissue-specific intervention may be critical to achieving desired therapeutic effects (Kwekkeboom et al., 2014).

## Data Availability Statement

All datasets presented in this study are included in the article/Supplementary Material.

## Ethics Statement

The animal study was reviewed and approved by Animal Ethical Experimentation Committee (Utrecht University). Written informed consent was obtained from the owners for the participation of their animals in this study.

## Author Contributions

ZL and JS conceived the study. ZL, JF, JD, and CM performed the experiment, collected and analyzed the data. ZL, JF, HA, PD, and JS wrote the manuscript. All authors listed have made a substantial, direct and intellectual contribution to the work, and approved it for publication.

## Conflict of Interest

The authors declare that the research was conducted in the absence of any commercial or financial relationships that could be construed as a potential conflict of interest.
